# Factors Associated With Early and Late Post-stroke Fatigue in Patients With Mild Impairment. Results From the Stroke Cohort Study Augsburg

**DOI:** 10.3389/fneur.2022.852486

**Published:** 2022-03-14

**Authors:** Inge Kirchberger, Florian Wallner, Jakob Linseisen, Philipp Zickler, Michael Ertl, Markus Naumann, Christine Meisinger

**Affiliations:** ^1^Chair of Epidemiology at the University Augsburg, University Hospital Augsburg, Augsburg, Germany; ^2^Institute for Medical Information Processing, Biometry and Epidemiology, Ludwig-Maximilians Universität Munich, Munich, Germany; ^3^Independent Research Group Clinical Epidemiology, Helmholtz Zentrum München, Neuherberg, Germany; ^4^Pettenkofer School of Public Health, Munich, Germany; ^5^Department of Neurology and Clinical Neurophysiology, University Hospital Augsburg, Augsburg, Germany

**Keywords:** stroke, fatigue, depression, disability, quality of life

## Abstract

**Background:**

Post-stroke fatigue is a common symptom after stroke. However, studies on the factors associated with early and late fatigue are scarce. The objective of this study was to identify variables associated with early and late fatigue.

**Methods:**

In the German Stroke Cohort Augsburg (SCHANA) study, participants were interviewed during their hospital stay and completed a postal questionnaire 3 and 12 months post-stroke. Fatigue was assessed using the Fatigue Assessement Scale (FAS). In addition, depression was measured by the Patient Health Questionnaire (PHQ-9), general health status by the EQ-5D visual analog scale, and physical activity by the International Physical Activity Questionnaire (IPAQ). Multivariable regression models were used to determine the associations between FAS scores at 3 and 12 months post-stroke and demographic, psychosocial and health-related covariables.

**Results:**

Among 505 participants, the frequency of fatigue was 31.1% 3 months and 29.1% 12 months post-stroke. Prior stroke (ß = 2.37, *p* = 0.0076), prior diagnosis of depression (ß = 5.04, *p* = 0.0001), higher NIHSS (ß = 0.25, *p* = 0.0360) and higher PHQ-9 scores (ß = 0.55, *p* < 0.0001) were significantly associated with higher fatigue levels 3 months post-stroke. Additionally, younger age (ß = −0.07, *p* = 0.0219), a worse rating of general health at baseline (ß = −0.04, *p* = 0.0287) and low pre-stroke physical activity (ß = −0.0004, *p* = 0.0089) were significantly associated with higher fatigue levels 12 months after stroke.

**Conclusions:**

Fatigue is a common and persisting symptom even in patients with mild impairment. Prior depressive disorder and early depressive symptoms were the most relevant predictors of both early and late fatigue.

## Introduction

Stroke is a major cause of disability and a wide range of mental, physical and behavioral impairments (1–4). Fatigue was found to be one of the most common symptoms after stroke with reported prevalences ranging between 25 and 85% depending on study design, time point and methods of assessment (5). Initially, fatigue was considered to be a symptom of post-stroke depression, but meanwhile scientific evidence supports that fatigue occurs also in persons without depression and led to the definition of a “post-stroke fatigue syndrome” (6).

Fatigue can be divided in early (up to 3 months post-stroke) and late (over 3 months post-stroke) fatigue and three patterns of the temporal course of fatigue can be described, namely persistent fatigue, recovered fatigue and late onset fatigue (7). Most longitudinal studies report consistent fatigue levels over 1–3 years (8–12) and early fatigue was shown to be a strong predictor of late fatigue in a few studies (13, 14).

However, the reasons, why patients develop fatigue are largely unknown. A number of factors which may be associated with fatigue have been discussed, such as age, gender, comorbid disorders, physical deconditioning, stroke characteristics, neurological or biological factors. So far, available study results on factors associated with fatigue are conflicting (7, 15–17). Moreover, it was hypothesized that the factors associated with early and late fatigue may be different (16). However, only a few studies with small sample sizes have addressed this topic and had controversial findings (13, 18). Thus, further methodologically sound studies are needed to clarify the possible determinants of both, early and late fatigue.

Consequently, the objectives of the present analysis were to determine the frequency of early and late fatigue and to identify variables associated with early and late fatigue in patients from the Stroke Cohort Augsburg (SCHANA).

## Materials and Methods

### Study Design

The present study uses data from the Stroke Cohort Augsburg (SCHANA), an observational single center cohort study, carried out as collaboration between the Chair of Epidemiology and the Department of Neurology and Clinical Neurophysiology at the University Hospital Augsburg. The cohort includes 945 patients aged 18 years and older with a confirmed ischemic or hemorrhagic stroke who were treated at the University Hospital Augsburg between September 2018 and November 2019.

After inclusion in the study, a standardized questionnaire was completed by the participants themselves or during a personal interview by a trained study nurse after the acute stroke event during their hospital stay. If the participant was not able to provide a self-report, proxy interviews were performed. Clinical data were extracted from the participants' medical record. Three and 12 months after the stroke event, the study participants received postal questionnaires for follow-up. Additional information about the SCHANA study can be found in the published study protocol (19).

The study was performed in accordance to the Declaration of Helsinki. Ethical approval was obtained from the ethics committee of the Ludwig-Maximilians-Universität München (Reference Number: 18-196). Written informed consent was obtained from all participants or legal caregivers.

### Survey Data

The baseline questionnaire included information on socio-demographics, disease history, and smoking behavior. Standardized questionnaires were used to assess physical activity, general health status and depressive symptoms. The follow-up questionnaires 3 and 12 months after the stroke event additionally contained questionnaires to assess fatigue and stroke-specific health-related quality of life (see Table 1).

**Table 1:** Summary of the standardized questionnaires used in the study.

**Questionnaire**	**Time points**	**Number of items**	**Score minimum–maximum**	**Interpretation**	**Classification**	**References**
Fatigue Assessment Scale (FAS)	3 and 12 months	10	10–50	Higher score = more fatigue	<24: no fatigue; 24–35: moderate fatigue; >35: high level of fatigue	(20–22)
Patient Health Questionnaire (PHQ-9)	Baseline, 3 and 12 months	9	0–27	Higher score = more symptoms of depression	<5: no depression; 5–10: mild depression;10–14: moderate depression; 15–19: moderately severe depression; 20–27: severe depression	(23–27)
International Physical Activity Questionnaire (IPAQ)	Baseline	7	0—not defined; Total metabolic equivalent time (MET)—minutes/week	Higher score = higher physical activity	–	(28–30)
European Quality of Life Questionnaire Visual Analog Scale (EQ-5D-VAS)	Baseline, 3 and 12 months	1	0–100	Higher score = better health status	–	(31, 32)
Stroke Impact Scale (SIS)	3 and 12 months	64	0–100	Higher score = better quality of life	–	(33–36)

### Clinical Data

Information on prior diseases and stroke severity collected by chart review were included in the present data analysis. A modification of the Charlson Comorbidity Index (mCCI) was built to assess multimorbidity (37). If more than one additional disease contained in a list of relevant diseases (e.g., hypertension, coronary heart disease, diabetes mellitus) was diagnosed, multimorbidity was ascertained (38, 39).

The degree of functional impairment post-stroke was assessed by the modified Rankin Scale (mRS), which ranges from zero (no symptoms) to six (dead). The mRS is deemed a valid and reliable instrument for stroke patients (40).

The National Institute of Health Stroke Scale (NIHSS) assesses stroke severity by the number of clinical symptoms after the stroke event. Higher total scores indicate more severe neurological symptoms (41, 42).

### Statistical Analysis

A descriptive analysis of the total sample characteristics stratified by the different fatigue levels was performed. Multiple linear regression models were used to determine associations between fatigue and a number of possible predictors and covariables. The covariables were selected according to the directed acyclic graph (DAG) method using the “DIA” diagram editor (Version 0.97.2). The DAG is a method to identify relevant covariables from literature and prior studies. The resulting DAG showed associations between fatigue and a number of covariables such as gender, age, prior stroke, multimorbidity, cardiovascular comorbidities, physical activity, general health status, depression, stroke severity and HRQOL.

The assumptions for multivariable linear regression analyses were tested using scatterplots to confirm linearity of associations, normal distribution of the residuals and homoscedasticity. Cook's distances and leverage diagnostic plots were used to check for leveraging outliers. Variance inflation factors were used to identify multicollinearity and Durbin Watson tests were calculated to describe autocorrelation. Interaction effects of age, gender, NIHSS, mRS and multimorbidity were tested, but were not statistically significant. For statistical tests an alpha level of 0.05 was defined. Statistical analyses were conducted using SAS® Version 9.4.

## Results

### Sample Characteristics

From the 945 participants of the SCHANA study, 585 (61.9%) responded to the survey 3 months post-stroke and 533 (56.4%) responded to the survey at 12 months. Participants with data available from both surveys (*n* = 505, 53.4%) were included in the present analysis.

Baseline information of participants who were excluded from this analysis due to missing follow-up surveys was compared with the included participants in order estimate a potential selection bias. Excluded participants were significantly older, less likely to be married and more likely to have a prior stroke or a prior diagnosis of depression than included participants. Significantly higher NHISS and mRS scores indicate that they had a more severe stroke. Moreover, excluded participants had higher PHQ-9 scores (indicating more depressive symptoms) and lower EQ5D-VAS scores (indicating a worse general health status) in comparison with those included in the present analysis.

Characteristics of the entire cohort can be found in Supplementary Table 1. Characteristics of the analysis sample are shown in Table 2. The study population consisted of 293 (58.0%) men and 212 (42.0%) women with a mean age of 68.7 ± 12.3 years. Most of them (*n* = 489, 96.8%) had an ischemic stroke and 404 (79.6%) had a first-ever-stroke. At admission the median NIHSS score was 1.0 and the median mRS score was 2.0. Multimorbidity was common in 78.8% of the participants, with hypertension being the most frequent concomitant disease in 81.4%. A prior diagnosis of a depressive disorder was documented in the medical chart in 50 (9.1%) of the participants.

**Table 2:** Sample characteristics, total sample and stratified by fatigue severity at 3 months post-stroke (*n*, %).

**Variable**	***n***	**Total (*n* = 505)**	**No fatigue^**a**^ (*n* = 348, 68.9%)**	**Moderate fatigue^**b**^ (*n* = 129, 25.5%)**	**Severe fatigue^**c**^ (*n* = 28, 5.6%)**	***P*-value^**d**^**
**Sociodemographic characteristics**						
Gender	505					
Male		293 (58.0)	212 (60.9)	65 (50.4)	16 (57.1)	0.1168
Female		212 (42.0)	136 (39.1)	64 (49.6)	12 (42.9)	
Age (in years), [mean (SD)]	505	68.7 (12.3)	69.3 (12.2)	67.44 (13.0)	66.64 (11.0)	0.0984
Married	505	338 (69.1)	235 (69.3)	83 (66.4)	20 (80.0)	0.4013
Education (>9 years)	505	208 (42.5)	147 (43.4)	53 (42.4)	8 (32.0)	0.5403
**Health-related characteristics**						
Ischemic stroke	505	489 (96.3)	339 (97.4)	124 (96.1)	26 (92.9)	0.3612
Hemorrhagic stroke	504	16 (3.2)	9 (2.6)	5 (3.9)	2 (7.1)	0.3639
Prior stroke	505	101 (20.4)	60 (17.2)	34 (26.4)	9 (32.1)	0.0255
Multimorbidity	505	398 (78.8)	272 (78.2)	107 (83.0)	19 (67.9)	0.1808
Diabetes mellitus	502	111 (22.1)	77 (22.3)	27 (20.9)	7 (25.0)	0.8830
Hypertension	505	411 (81.4)	281 (80.8)	110 (85.3)	20 (71.4)	0.2007
Smoking	505					0.8205
Current smoker		65 (12.9)	42 (12.1)	19 (14.7)	4 (14.2)	
Ex-smoker		219 (43.4)	148 (42.5)	59 (45.8)	12 (42.9)	
Never smoker		221 (43.7)	158 (45.4)	51 (39.5)	12 (42.9)	
Prior diagnosis of depressive disorder	505					<0.0001
Yes		50 (9.9)	20 (5.8)	21 (16.3)	9 (32.2)	
No		186 (36.8)	138 (39.7)	42 (32.6)	6 (21.4)	
No information		269 (53.3)	190 (54.6)	66 (51.1)	46 (46.4)	
Stroke severity
NIHSS admission [median (Q1; Q2)]	494	1.0 (0.0; 4.0)	1.0 (0.0; 3.0)	2.0 (1.0; 4.0)	4.0 (1.0; 8.0)	0.0022
NIHSS discharge [median (Q1; Q2)]	441	0.0 (0.0; 1.0)	0.0 (0.0; 1.0)	0.0 (0.0; 1.0)	1.0 (0.0; 5.0)	0.0025
mRS admission [median (Q1; Q2)]	495	2.0 (1.0; 3.0)	2.0 (1.0; 3.0)	2.0 (1.0; 3.0)	3.5 (2.0; 4.0)	0.0034
mRS discharge [median (Q1; Q2)]	493	1.0 (0.0; 2.0)	1.0 (0.0; 2.0)	1.0 (0.0; 2.0)	2.0 (0.0; 4.0)	0.0039
Symptoms of depression (PHQ-9) [mean (SD)]	478	4.7 (4.2)	3.7 (3.4)	6.8 (4.6)	8.0 (5.9)	<0.0001
General health status (EQ-5D VAS) [mean (SD)]	489	62.8 (21.1)	66.0 (20.1)	56.6 (21.0)	50.0 (23.0)	<0.0001
Physical activity (IPAQ Total MET-minutes/week) [mean (SD)]	431	2,330.8 (2827.2)	2,443.0 (2928.5)	2,146.7 (2594.0)	1,686.8 (2446.9)	0.1543

a *Fatigue Assessment Scale score < 24*.

b *Fatigue Assessment Scale score 24–35*.

c *Fatigue Assessment Scale score > 35*.

d *Test of differences between no, moderate and severe fatigue*.

*NIHSS, National Institute of Health Stroke Scale; mRS, Modified Rankin Scale; PHQ, Patient Health Questionnaire; EQ-5D VAS, EuroQol 5D Questionnaire, Visual Analog Scale; IPAQ, International Physical Activity Questionnaire; MET, Metabolic Equivalent Time*.

Participants with more severe fatigue were significantly more likely to have had a prior stroke, a prior diagnosis of a depressive disorder, higher NIHSS and mRS scores at admission and discharge, more depressive symptoms (PHQ-9) and a worse general health status (EQ-5D VAS) than participants with less severe fatigue (see Table 2).

### Frequency of Early and Late Fatigue

Three months post-stroke, 31.1% of the participants reported to have moderate (25.6%) or severe (5.5%) fatigue (see Table 3). After 12 months the proportion of participants with fatigue was 29.1%, with 22.4% reporting moderate fatigue and 6.7% with a severe level of fatigue. Considering the temporal course, in most of the participants with fatigue at 3 months (*n* = 157) it persisted from 3 to 12 months (*n* = 96), whereas *n* = 61 recovered. Late onset fatigue 12 months after stroke was present in 51 participants (10.1% of all participants).

**Table 3:** Frequency of fatigue 3 and 12 months post-stroke [*n* (%)].

	**12 months post-stroke**			
**3 months post-stroke**	**No fatigue^**a**^**	**Moderate fatigue^**b**^**	**Severe fatigue^**c**^**	**Total**
No fatigue	297 (58.8%)	50 (9.9%)	1 (0.2%)	348 (68.9%)
Moderate fatigue	58 (11.5%)	56 (11.1%)	15 (3.0%)	129 (25.6%)
Severe fatigue	3 (0.6%)	7 (1.4%)	18 (3.6%)	28 (5.5%)
Total	358 (70.9%)	113 (22.4%)	34 (6.7%)	505 (100%)

a *Fatigue Assessment Scale score < 24*.

b *Fatigue Assessment Scale score 24–35*.

c *Fatigue Assessment Scale score > 35*.

### Baseline Variables Associated With Fatigue 3 and 12 Months Post-stroke

The multivariable linear regression model of the FAS score 3 months post-stroke is shown in Table 4. Independent variables were gender, age, prior stroke, NIHSS and mRS scores at admission, multimorbidity, prior depressive disorder, PHQ-9, EQ-5D VAS and IPAQ total MET-minutes/week assessed at baseline. The variables explained 17% (adjusted *R*^2^) of the FAS variance. Prior stroke was significantly (ß = 2.37, *p* = 0.0076) related with more fatigue, as well as higher NIHSS scores at admission (ß = 0.25, *p* = 0.0360). The absence of a prior diagnosis of a depressive disorder (ß = −5.04, *p* = 0.0001) and lacking information on a prior diagnosis of a depressive disorder in the medical chart (ß = −3.83, p = 0.0027) were both significantly associated lower FAS scores indicating lower fatigue levels. In addition, higher PHQ-9 scores indicating more symptoms of depression, were significantly (= 0.55, *p* < 0.0001) associated with a higher level of fatigue.

**Table 4:** Multivariable linear regression model of fatigue (Fatigue Impact Scale score) 3 and 12 months post-stroke (*n* =422).

		**3 months post-stroke**			**12 months post-stroke**		
**Variable**	**Reference**	**Beta**	**95% CI**	***p*-value**	**Beta**	**95% CI**	***p*-value**
Intercept		25.80	19.47; 32.13	<0.0001	25.41	18.83; 31.99	<0.0001
Gender (male)	Female	0.17	−1.25; 1.59	0.8128	0.32	−1.15; 1.80	0.6655
Age		−0.05	−0.11; 0.01	0.0899	−0.07	−0.14; 0.01	0.0219
Prior stroke (yes)	No	2.37	0.63; 4.10	0.0076	2.77	0.97; 4.57	0.0026
NIHSS at admission		0.25	0.02; 0.49	0.0360	0.27	0.03; 0.52	0.0279
mRS score 1	Score 0	−1.37	−3.77; 1.02	0.2603	−0.32	−2.81; 2.17	0.8010
mRS score 2	Score 0	−0.56	−2.75; 1.63	0.6172	1.72	−0.56; 4.00	0.1380
mRS score 3	Score 0	−0.08	−2.49; 2.33	0.9491	0.47	−2.04; 2.98	0.7131
mRS scores 4 + 5	Score 0	−1.17	−3.88; 1.53	0.3933	1.15	−1.66; 3.96	0.4226
Multimorbidity (yes)	No	0.33	−1.39; 2.05	0.7094	1.58	−0.20; 3.37	0.0819
Prior depressive disorder (no)	Yes	−5.04	−7.59; −2.49	0.0001	−3.35	−6.00; −0.70	0.0135
Prior depressive disorder (no information)	Yes	−3.83	−6.32; −1.33	0.0027	−3.05	−5.64; −0.46	0.0212
Symptoms of depression (PHQ-9)		0.55	0.37; 0.72	<0.0001	0.63	0.45; 0.82	<0.0001
General health status (EQ-5D VAS)		−0.01	−0.05; 0.02	0.4704	−0.04	−0.08; 0.01	0.0287
Physical activity (IPAQ Total MET-minutes/week)		−0.0001	−0.0003; 0.0001	0.3263	−0.0004	−0.0006; 0.00009	0.0089

*CI, Confidence interval; NIHSS, National Institute of Health Stroke Scale; mRS, Modified Rankin Scale, reference: score 0 = no symptoms; higher scores indicate higher severity; PHQ, Patient Health Questionnaire; EQ-5D VAS, EuroQol 5D Questionnaire, Visual Analog Analogue Scale; IPAQ, International Physical Activity Questionnaire; MET, Metabolic Equivalent Time*.

The regression model of the FAS score 12 months post-stroke showed that the independent baseline variables explained 23% (adjusted *R*^2^) of the FAS variance. Similar to the regression model for 3 months post-stroke, prior stroke, NIHSS at admission, prior depressive disorder and PHQ-9 were significantly associated with the FAS score (see Table 4). In addition, younger age (ß = −0.07, *p* = 0.0219), a worse rating of general health at baseline (ß = −0.04, *p* = 0.0287) and low pre-stroke physical activity (ß = −0.0004, *p* = 0.0089) were significantly associated with higher fatigue levels.

When adding the FAS score at 3 months as an additional independent variable to the model, the explained variance increased to 38% (adjusted *R*^2^). The FAS score at 3 months was significantly associated with the FAS at 12 months (ß = 0.46, *p* < 0.0001) (see Supplementary Table 2).

In the regression models NIHSS and mRS at admission were replaced by the same variables at discharge in order to check for any differences. However, the results did not change. NIHSS remained significant, whereas mRS did not. Thus, the information from admission was included in the final models.

Since the PHQ-9 includes one item requesting tiredness, the regression models were additionally calculated with a PHQ score which excludes this item. Both scores were strongly correlated (Pearson *r* = 0.98, *p* < 0.0001). The results of the regression models were similar (see Supplementary Table 3). The association of the modified PHQ with fatigue was also highly significant (3 months: ß = 0.59, *p* < 0.0001; 12 months: ß = 0.72, *p* < 0.0001).

### Cross-Sectional Associations With Fatigue 3 and 12 Months Post-stroke

Table 5 shows the significant association between dichtomized FAS and PHQ scores (fatigue yes/no, depression yes/no). A total of 14.8% (3 months) and 16.3% (12 months) of the participants have both depression and fatigue. Three months post-stroke, participants with depression had a 30.91-fold odds [95% confidence interval (CI) 15.31–62.41] to also have moderate to severe levels of fatigue, compared to participants without depression. Twelve months post-stroke, the odds were still high with an odds ratio of 21.32 (95% CI 12.22–37.18). However, among the participants with fatigue, 44.2% (3 months) and 52.2% (12 months) did not reach PHQ-9 scores indicating a depressive disorder.

**Table 5:** Associations between presence of fatigue and depressiveness 3 and 12 months post-stroke.

	**FAS**		
**PHQ-9**	**No fatigue^**a**^**	**Fatigue^**b**^**	**Total**
**3 months post-stroke**			
No depression^c^	338 (66.9%)	82 (16.3%)	420 (83.2%)
Depression^d^	10 (2.0%)	75 (14.8%)	85 (16.8%)
Total	348 (68.9%)	157 (31.1%)	505 (100%)
**12 months post-stroke**			
No depression	338 (66.9%)	65 (12.8%)	403 (79.8%)
Depression	20 (4.0%)	82 (16.3%)	102 (20.2%)
Total	358 (70.9%)	147 (29.1%)	505 (100%)

a *Score < 24*.

b *Score ≥ 24*.

c *Score ≤ 9*.

d *Score >9*.

*FAS, Fatigue Assessment Scale; PHQ-9, Patient Health Questionnaire 9 Item Version*.

In the cross-sectional regression models, SIS, PHQ-9 and EQ-5D VAS measured at 3 or 12 months, respectively, were included as independent variables. The regression model of fatigue at 3 months post-stroke showed that higher PHQ-9 scores (ß = 0.68, *p* < 0.0001) indicating more depressive symptoms and lower EQ-5D VAS scores (ß = −0.06, *p* = 0.0203) indicating a worse health status were significantly and independently associated with higher fatigue levels (see Table 6). Similarly, these variables were significant in the model calculated for fatigue at 12 months post-stroke. Additionally, in the 12 months-model a significant association between communication problems (SIS) and fatigue was found (ß = −0.14, *p* = 0.007).

**Table 6:** Multivariable linear regression model of fatigue (Fatigue Impact Scale score) at 3 and 12 months post-stroke (*n* =448).

	**3 months post-stroke (adjusted *R*^2^ = 0.33)**			**12 months post-stroke (adjusted *R*^2^ = 0.52)**		
**Variable**	**Beta**	**95% CI**	***p*-value**	**Beta**	**95% CI**	***p*-value**
SIS communication	−0.08	−0.16; 0.002	0.0553	−0.14	−0.22; −0.06	0.0007
SIS memory/cognition	−0.01	−0.09; 0.06	0.7145	−0.03	−0.10; 0.04	0.3800
SIS emotion	−0.003	−0.06; 0.05	0.9205	−0.03	−0.08; 0.02	0.2207
SIS participation	−0.02	−0.07; 0.02	0.2988	0.02	−0.02; 0.05	0.3889
SIS physical	0.05	−0.02; 0.11	0.2020	0.01	−0.05; 0.07	0.8080
Symptoms of depression (PHQ-9)	0.68	0.42; 0.93	<0.0001	0.70	0.49; 0.90	<0.0001
General health status (EQ-5D VAS)	−0.06	−0.11; 0.01	0.0203	−0.07	−0.11; 0.03	0.0011

*CI, Confidence interval; PHQ, Patient Health Questionnaire; EQ-5D VAS, EuroQol 5D Questionnaire, Visual Analog Scale*.

*Models were adjusted for age, gender, prior stroke, prior diagnosis of depression and NIHSS at admission*.

Exclusion of the PHQ from the models changed the results. In the regression model for fatigue at 3 months post-stroke, the SIS domains communication (ß = −0.12, *p* = 0.0036), emotion (ß = −0.08, *p* = 0.0033) and participation (ß = −0.05, *p* = 0.0350) were significantly associated with the level of fatigue. Similarly, in the regression model at 12 months post-stroke the SIS domains communication (ß = −0.16, *p* = 0.0001) and emotion (ß = −0.11, *p* < 0.0001) emerged as significantly related variables.

## Discussion

The present study showed that about one third of participants experienced fatigue 3 or 12 months after the stroke event. This frequency is at the bottom of the range between 25 and 85% reported by a meta-analysis (5). However, since the present study sample mainly consisted of patients with mild impairment, the prevalence of fatigue among these patients seems alarmingly high. Moreover, the present study confirmed that most of the patients have persistent fatigue and that early fatigue is a strong predictor of late fatigue (11, 13). Depression (prior diagnosis of a depressive disorder, high baseline PHQ-9 scores) was the main contributor to higher levels of fatigue 3 and 12 months post-stroke, besides prior stroke and higher NIHSS scores. Additionally, younger age, a worse rating of general health at baseline and low pre-stroke physical activity were significantly associated with higher fatigue levels 12 months after stroke.

Two predictors related to depression (high baseline PHQ-9 and prior depressive disorder) were strongly associated with fatigue in our sample. Two previous systematic reviews including 98 (17) and 35 observational studies (43), and a narrative review (16) have reported a similar relationship between depression and fatigue after stroke. Since some of the instruments to measure depression also include items assessing fatigue, Wu et al. (43) performed a sensitivity analysis on studies using instruments without fatigue items, but the results were not significantly different. The same was found in the sensitivity analyses of the present study, when eliminating the fatigue item from the PHQ-9 score. The role of a prior depressive disorder was only considered in a few studies so far. Consistent with the present study, Naess et al. (9) found that pre-stroke depression was significantly related with fatigue, whereas Snaphaan et al. (13) did not.

So far, there is no consistent evidence that neurological stroke characteristics were related with fatigue (16, 17). Prior stroke was significantly associated with fatigue in only four out of 28 studies included in a systematic review of 98 observational studies (17). The small numbers of patients with prior stroke in these studies may have contributed to this lack of consistency. Similarly, a review indicated no significant association of stroke severity and fatigue in 8 out of 10 published studies (7). Two studies found an association of stroke severity at admission and fatigue 6 months after stroke, however, confounding effects of depression and anxiety were not considered (18, 44). In contrast, the present study demonstrated that higher NIHSS scores at admission were significantly and independently associated with higher levels of fatigue 3 and 12 months post-stroke.

Of interest, the finding that pre-stroke physical activity was inversely related with fatigue 12 months post-stroke has not been reported so far. However, it is in line with other results from the SCHANA study, which showed that pre-stroke physical activity was significantly associated with physical HRQOL 3 months post-stroke (45).

It is essential to mention, that the variance of the FAS score explained by the predictors ranged between 17 and 23%. Thus, additional factors seem to contribute to the development and maintenance of fatigue. Biological factors such as inflammatory processes may play an important role specifically for the development of early fatigue, but have not been extensively studied (46). In the present study the psychosocial and clinical variables included, explained more variance of late fatigue than of early fatigue and additional factors such as younger age, pre-stroke physical activity and early post-stroke general health status significantly determined late fatigue but not early fatigue. These findings may support the hypothesis, that early fatigue is triggered by biological factors whereas psychosocial factors and post-stroke impairments contribute to the maintenance of late fatigue (16, 43). The determination of predictors which contribute to the development of early and late fatigue is essential since it enables an early identification of persons who are at risk of developing fatigue. Early screening for risk factors, specifically for prior or current depression and physical inactivity, by health care professionals in the rehabilitation setting may facilitate an early start of interventions. Similar to post-stroke depression (47) studies providing sufficient evidence as to whether a screening for fatigue can reduce patient burden or improve outcomes and what appropriate instruments to assess fatigue are, are missing so far. Moreover, the role of biological processes which seem to trigger the development of early fatigue is still underinvestigated and should accompany research on psychosocial factors that contribute to long-term fatigue.

A major strength of the present study is its large sample size and the prospective longitudinal design which enabled an analysis of both early and late fatigue. A number of relevant covariables, which have been selected by methodologically sound techniques, were included in the multivariable analyses. Some of them, e.g., pre-stroke depression diagnosis and physical activity, were rarely considered in previous studies. Fatigue was assessed using a psychometrically well-tested and sound instrument.

However, some limitations should be considered. First, study data was derived from a single university hospital in Southern Germany, which may limit external validity. Second, there is an underrepresentation of severely affected participants in the study sample, since a number of hospitalized patients declined their participation and 47% of the study participants did not respond to the follow-up surveys. Participants who were lost to follow-up might also have high levels of fatigue. Thus, the prevalence of fatigue in the study sample might have been underestimated. Although it is unclear how many participants were not able to respond to the questions themselves, it is expected that most of the participants were able to complete the questionnaires, since the sample was characterized by mild impairments. However, the use of proxy data might have led to an overestimation of fatigue and other subjective impairments (48). Furthermore, the PHQ-9 questionnaire used to assess depression contained one item on fatigue, which might have distorted the association between depression and fatigue. However, sensitivity analyses did not reveal any biases.

In summary, the present study showed that fatigue is a common and persisting symptom even in participants with mild impairment. Prior depressive disorder and early depressive symptoms were the most relevant predictors of both early and late fatigue. Since prior depression was rarely considered in previous studies, it seems important to include it in future trials. However, the study also indicated that different factors may contribute to the development of early vs. late fatigue. Thus, further studies are needed to increase the knowledge about contribution and interplay of biological and psychosocial factors for the development of early and late fatigue.

## Data Availability Statement

The raw data supporting the conclusions of this article will be made available by the authors, without undue reservation.

## Ethics Statement

The studies involving human participants were reviewed and approved by Ethics Committee of the Ludwig-Maximilians-Universität München (Reference Number: 18-196). The patients/participants provided their written informed consent to participate in this study.

## Author Contributions

IK: conceptualization, methodology, formal analysis, and writing—original draft. FW: formal analysis and writing—original draft. JL and MN: conceptualization, resources, supervision, and writing—review and editing. PZ and ME: conceptualization, investigation, and writing—review and editing. CM: conceptualization, investigation, supervision, and writing—review and editing. All authors contributed to the article and approved the submitted version.

## Conflict of Interest

The authors declare that the research was conducted in the absence of any commercial or financial relationships that could be construed as a potential conflict of interest.

## Publisher's Note

All claims expressed in this article are solely those of the authors and do not necessarily represent those of their affiliated organizations, or those of the publisher, the editors and the reviewers. Any product that may be evaluated in this article, or claim that may be made by its manufacturer, is not guaranteed or endorsed by the publisher.
