# Supplementary material for: Factors Associated With Early and Late Post-stroke Fatigue in Patients With Mild Impairment. Results From the Stroke Cohort Study Augsburg
Source: Front Neurol. 2022 Mar 14;13:852486. doi: 10.3389/fneur.2022.852486 (PMC8964017; doi:10.3389/fneur.2022.852486)
Supplement: Supplementary file 1 [file Table_1.DOCX]

Supplementary Table 1: Characteristics of the total cohort (n=945)

| Variable | n | Total  (n=945) |
| --- | --- | --- |
| **Sociodemographic characteristics** |  |  |
| Gender | 945 |  |
| Male |  | 531 (56.2) |
| Female |  | 414 (43.8) |
| Age (in years), [*mean (SD)*] | 945 | 69.4 (13.1) |
| Married | 787 | 488 (62.0) |
| Education ( > 9 years) | 785 | 323 (41.2) |
|  |  |  |
| **Health-related characteristics** |  |  |
| Ischemic stroke | 945 | 908 (96.1) |
| Haemorrhagic stroke | 943 | 34 (3.6) |
| Prior stroke | 945 | 160 (16.9) |
| Multimorbidity | 945 | 723 (76.5) |
| Diabetes mellitus | 936 | 209 (22.3) |
| Hypertension | 945 | 764 (81.9) |
| Smoking | 945 |  |
| Current smoker |  | 125 (13.2) |
| Ex-smoker |  | 344 (36.4) |
| Never smoker |  | 476 (50.4) |
| Prior diagnosis of depressive disorder | 944 |  |
| Yes |  | 118 (12.5) |
| No |  | 347 (36.8) |
| No information |  | 479 (50.7) |
|  |  |  |
| **Stroke severity** |  |  |
| NIHSS^4^ admission *[median (Q1;Q2)]* | 928 | 2.0 (0.0;4.0) |
| NIHSS^4^ discharge *[median (Q1;Q2)]* | 834 | 0.0 (0.0;2.0) |
| mRS^5^ admission *[median (Q1;Q2)]* | 931 | 2.0 (1.0;4.0) |
| mRS^5^ discharge *[median (Q1;Q2)]* | 930 | 1.0 (0.0;2.0) |
|  |  |  |
| Symptoms of depression (PHQ-9^6^) *[mean (SD)]* | 754 | 5.1 (4.4) |
| General health status (EQ-5D VAS^7^) *[mean (SD)]* | 784 | 60.1 (22.3) |
| Physical activity (IPAQ Total MET-minutes/week^8^) *[mean (SD)]* | 675 | 2450.3 (2934.9) |
|  |  |  |

^1^ Fatigue Assessment Scale score < 24; ^2^ Fatigue Assessment Scale score 24 – 35; ^3^ Fatigue Assessment Scale score > 35; ^4^ National Institute of Health Stroke Scale; ^5^ Modified Rankin Scale, ^6^ Patient Health Questionnaire; ^7^ EuroQol 5D Questionnaire, Visual Analogue Scale; ^8^ International Physical Activity Questionnaire, Metabolic Equivalent Time (MET);
