# Supplementary material for: Factors Associated With Early and Late Post-stroke Fatigue in Patients With Mild Impairment. Results From the Stroke Cohort Study Augsburg
Source: Front Neurol. 2022 Mar 14;13:852486. doi: 10.3389/fneur.2022.852486 (PMC8964017; doi:10.3389/fneur.2022.852486)
Supplement: Supplementary file 2 [file Table_2.DOCX]

**Supplementary Table 2:** Multivariable linear regression model of fatigue (Fatigue Impact Scale score) 12 months post stroke (n =422) with 3 months FAS score added as predictor. Adjusted R^2^=0.38

| **Variable** | **Reference** | **Beta** | **95% CI^1^** | **p-value** |
| --- | --- | --- | --- | --- |
| Intercept |  | 13.64 | 7.27; 20.01 | <.0001 |
| Gender (male) | female | 0.25 | -1.08; 1.57 | 0.7151 |
| Age |  | -0.05 | -0.11; 0.01 | 0.0857 |
| Prior stroke (yes) | no | 1.70 | 0.06; 3.33 | 0.0420 |
| NIHSS^2^ at admission |  | 0.17 | -0.06; 0.38 | 0.1566 |
| mRS^3^ score 1 | Score 0 | 0.31 | -1.94; 2.55 | 0.7881 |
| mRS^3^ score 2 | Score 0 | 1.98 | -0.07; 4.02 | 0.0588 |
| mRS^3^ score 3 | Score 0 | 0.51 | -1.75; 2.76 | 0.6601 |
| mRS^3^ scores 4+5 | Score 0 | 1.68 | -0.85; 4.21 | 0.1917 |
| Multimorbidity (yes) | no | 1.44 | -0.17; 3.04 | 0.0798 |
| Prior depressive disorder (no) | yes | -1.05 | -3.48; 1.38 | 0.3972 |
| Prior depressive disorder  (no information) | yes | -1.30 | -3.66; 1.05 | 0.2778 |
| Symptoms of depression (PHQ-9^4^) |  | 0.38 | 0.21; 0.56 | <.0001 |
| General health status (EQ-5D VAS^5^) |  | -0.04 | -0.07; 0.002 | 0.0151 |
| Physical activity (IPAQ Total MET-minutes/week^6^) |  | -0.0003 | -0.0005; 0.00006 | 0.0151 |
| Fatigue (FAS 3 months assessment) |  | 0.46 | 0.37; 0.55 | <.0001 |

^1^ Confidence interval; ^2^ National Institute of Health Stroke Scale; ^3^ Modified Rankin Scale, reference: score 0 = no symptoms; higher scores indicate higher severity; ^4^ Patient Health Questionnaire; ^5^ EuroQol 5D Questionnaire, Visual Analogue Scale; ^6^ International Physical Activity Questionnaire, Metabolic Equivalent Time (MET)
