# Supplementary material for: Factors Associated With Early and Late Post-stroke Fatigue in Patients With Mild Impairment. Results From the Stroke Cohort Study Augsburg
Source: Front Neurol. 2022 Mar 14;13:852486. doi: 10.3389/fneur.2022.852486 (PMC8964017; doi:10.3389/fneur.2022.852486)
Supplement: Supplementary file 3 [file Table_3.DOCX]

**Supplementary Table 3:** Multivariable linear regression model of fatigue (Fatigue Impact Scale score) 3 months and 12 months post stroke (n =422)

|  |  | **3 months post stroke** | | | **12 months post stroke** | | |
| --- | --- | --- | --- | --- | --- | --- | --- |
| **Variable** | **Reference** | **Beta** | **95% CI^1^** | **p-value** | **Beta** | **95% CI^1^** | **p-value** |
| Intercept |  | 26.66 | 20.32; 33.00 | <.0001 | 26.10 | 19.52; 32.67 | <.0001 |
| Gender (male) | female | 0.26 | -1.17; 1.68 | 0.7217 | 0.42 | -1.06; 1.90 | 0.5795 |
| Age |  | -0.06 | -0.12; 0.01 | 0.0779 | -0.08 | -0.14; 0.01 | 0.0190 |
| Prior stroke (yes) | no | 2.32 | 0.58; 4.07 | 0.0092 | 2.74 | 0.93; 4.55 | 0.0031 |
| NIHSS^2^ at admission |  | 0.24 | 0.01; 0.48 | 0.0444 | 0.27 | 0.02; 0.51 | 0.0349 |
| mRS^3^ score 1 | Score 0 | -1.36 | -3.77; 1.05 | 0.2673 | -0.29 | -2.79; 2.21 | 0.8181 |
| mRS^3^ score 2 | Score 0 | -0.62 | -2.82; 1.59 | 0.5828 | 1.68 | -0.61; 3.97 | 0.1495 |
| mRS^3^ score 3 | Score 0 | -0.18 | -2.62; 2.25 | 0.8812 | 0.36 | -2.16; 3.97 | 0.7774 |
| mRS^3^ scores 4+5 | Score 0 | -1.18 | -3.91; 1.54 | 0.3929 | 1.14 | -1.68; 3.97 | 0.4269 |
| Multimorbidity (yes) | no | 0.32 | -1.41; 2.05 | 0.7159 | 1.55 | -0.24; 3.35 | 0.0899 |
| Prior depressive disorder (no) | yes | -5.18 | -7.74; -2.61 | <.0001 | -3.43 | -6.10; -0.77 | 0.0116 |
| Prior depressive disorder  (no information) | yes | -4.02 | -6.52; -1.51 | 0.0017 | -3.20 | -5.80; -0.61 | 0.0157 |
| Symptoms of depression (PHQ-8^4^) |  | 0.59 | 0.39; 0.81 | <.0001 | 0.72 | 0.51; 0.94 | <.0001 |
| General health status (EQ-5D VAS^5^) |  | -0.02 | -0.05; 0.02 | 0.3748 | -0.04 | -0.08; 0.01 | 0.0207 |
| Physical activity (IPAQ Total MET-minutes/week^6^) |  | -0.0001 | -0.0003; 0.0001 | 0.3335 | -0.0004 | -0.0006; 0.00009 | 0.0089 |

^1^ Confidence interval; ^2^ National Institute of Health Stroke Scale; ^3^ Modified Rankin Scale, reference: score 0 = no symptoms; higher scores indicate higher severity; ^4^ Patient Health Questionnaire 8 Items excluding fatigue item ^5^ EuroQol 5D Questionnaire, Visual Analogue Scale; ^6^ International Physical Activity Questionnaire, Metabolic Equivalent Time (MET)
